# Targeting the miR-200c/LIN28B axis in acquired EGFR-TKI resistance non-small cell lung cancer cells harboring EMT features

**DOI:** 10.1038/srep40847

**Published:** 2017-01-13

**Authors:** Hiroki Sato, Kazuhiko Shien, Shuta Tomida, Kazuhiro Okayasu, Ken Suzawa, Shinsuke Hashida, Hidejiro Torigoe, Mototsugu Watanabe, Hiromasa Yamamoto, Junichi Soh, Hiroaki Asano, Kazunori Tsukuda, Shinichiro Miyoshi, Shinichi Toyooka

**Affiliations:** 1 Departments of General Thoracic Surgery and Breast and Endocrinological Surgery, Okayama University Graduate School of Medicine, Dentistry and Pharmaceutical Sciences, Okayama, Japan; 2 Departments of Bioinformatics, Okayama University Graduate School of Medicine, Dentistry and Pharmaceutical Sciences, Okayama, Japan; 3 Departments of Clinical Genomic Medicine, Okayama University Graduate School of Medicine, Dentistry and Pharmaceutical Sciences, Okayama, Japan

## Abstract

MicroRNA (miR)-200 family members (miR-200s) are frequently silenced in advanced cancer and have been implicated in the process of epithelial-to-mesenchymal transition (EMT). We previously reported that miR-200s were silenced through promoter methylation in acquired EGFR-tyrosine kinase inhibitor (TKI) resistant non-small cell lung cancer (NSCLC) cells harboring EMT features. In this study, we examined the functional role of miR-200s in NSCLC cells and investigated a novel approach to overcoming acquired EGFR-TKI resistance. In the analysis of NSCLC cell lines, each of the miR-200s expression-silenced cell lines showed promoter methylation. Significant correlations between miR-200c silencing and several oncogenic pathway alterations, including EMT-changes and LIN28B overexpression, were observed in the database analysis. In addition, *EGFR*-wild type cell lines had lower miR-200s expression levels than *EGFR*-mutant cell lines. The introduction of miR-200c using pre-miR-200c caused LIN28B suppression in cells with acquired EGFR-TKI resistance that harbored EMT features. Interestingly, both the introduction of miR-200c and the knockdown of LIN28B produced an antitumor effect in acquired EGFR-TKI resistance cells, whereas these manipulations were not effective in parental cells. The miR-200c/LIN28B axis plays an important role in cells with acquired resistance to EGFR-TKI that harbor EMT features and might be a useful therapeutic target for overcoming resistance.

The prognosis of advanced non-small cell lung cancer (NSCLC) patients remains unsatisfactory, despite improvements in diagnosis and therapeutic strategies. A considerable number of advanced NSCLC patients are not sensitive to treatment, and their tumors frequently develop drug resistance, usually leading to a relapse and cancer death. In 2004, mutations in the epidermal growth factor receptor (EGFR) gene that cause oncogene addiction to EGFR were discovered in NSCLC^1^,^2^. EGFR-tyrosine kinase inhibitors (TKIs) have exhibited significant effects against NSCLC with EGFR mutations^3^,^4^,^5^, however, patients who initially respond to EGFR-TKIs eventually acquire resistance, which is a critical problem in the treatment of patients with advanced NSCLC. Several mechanisms are believed to be responsible for acquired EGFR-TKIs resistance. Secondary *EGFR* T790M and *MET* amplification may together account for 70% of this resistance, and activation of the MET/HGF axis, leading to the acquisition of an epithelial-to-mesenchymal transition (EMT) signature, and transformation from NSCLC into small cell lung cancer have also been reported as possible mechanisms of acquired resistance to EGFR-TKIs in NSCLC^6^,^7^,^8^,^9^,^10^,^11^. Among these mechanisms, an EMT is negatively associated with EGFR-TKI sensitivity in NSCLC^12^,^13^. The activation of several pathways and molecules, including TGF-β-IL-6, Slug, Notch-1, PDGFR, ZEB1, Axl, and MED12, is reportedly associated with acquired EGFR-TKI resistance in cells with EMT features^14^. In addition, we previously reported a relation between epigenetic alteration related to EMT and EGFR-TKI resistance^15^. We have also demonstrated the CpG island hypermethylation-associated silencing of microRNA (miR)-200 family members in acquired EGFR-TKI resistance cells with EMT features. However, the detailed mechanisms underlying the EMT-related acquired resistance to EGFR-TKIs remains unclear.

MicroRNAs are small noncoding, endogenous, single-stranded RNAs that are commonly deregulated in human cancers. Several miRs show abnormal expression patterns in cancers with consequent alteration of the target oncogenes or tumor suppressor genes^16^. MicroRNA profiling for NSCLC has been previously conducted by several groups to predict patient survival^17^,^18^,^19^. These profiles have also been correlated with clinicopathological parameters of NSCLC patients^20^,^21^. Bishop *et al*. used this approach for classification of NSCLC^22^. Among such miRs, miR-200 family members (miR-200s: miR-200a, 200b, 200c, 141, and 429) have been reported to play important roles in the progression of NSCLC^23^,^24^. MiR-200s are genetically grouped in two polycistronic units: miR-200b/200a/429 (miR-200ba429) and miR-200c/141 (miR-200c141), clustered in chromosomes 1 and 12, respectively^24^. They are frequently silenced in advanced cancer and have been implicated in EMT and tumor invasion through the targeting of transcriptional repressors of E-cadherin, ZEB1 and ZEB2^25^. ZEB1 is also known to repress miR-200s transcription in a negative feedback loop. MiR-200c silencing was also reported in cancer stem cells (CSCs), suggesting that miR-200c deregulation is a key event in multiple levels of tumor biology^26^.

Several of the miRs identified from these studies have been associated with key regulatory pathways including EGFR and KRAS in NSCLC. In this study, we examined the functional role of miR-200s in NSCLC cell lines, and investigated the mechanisms underlying the EMT and acquired EGFR-TKI resistance to develop a novel approach for overcoming acquired resistance to EGFR-TKIs in NSCLC.

## Results

### Expression and methylation statuses of miR-200s in NSCLC

**Figure 1:**
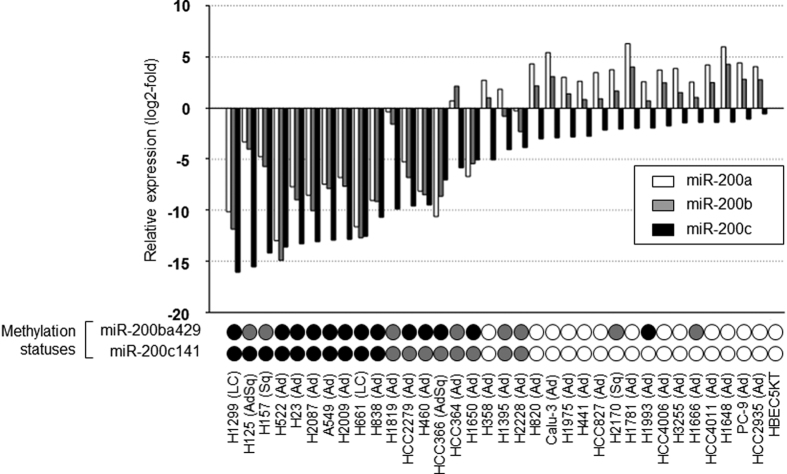
Expression and methylation statuses of miR-200s in 34 NSCLC cell lines and HBEC-5KT. The miR-200a, miR-200b, and miR-200c expression statuses as determined using qRT-PCR and the miR-200ba429 and miR-200c141 methylation statuses as determined using methylation-specific PCR (MSP) in 34 NSCLC cell lines and HBEC-5KT are shown. The miR-200s expression levels in HBEC-5KT were set at 1, and the expression levels in the NSCLC cells were shown relative to those in the HBEC-5KT cell line. For the MSP assay, each circle represents the promoter methylation status (white circle, unmethylated; gray circle, partially methylated; black circle, methylated).

The miR-200s expression statuses determined using quantitative reverse transcription-PCR (qRT-PCR) and the methylation statuses determined using methylation-specific PCR (MSP) in 34 NSCLC cell lines and the bronchial epithelial cell line HBEC-5KT are shown in Fig. 1. MiR-200ba429 and miR-200c141 were unmethylated in HBEC-5KT, and the miR-200s expression level in HBEC-5KT was used as a control. In the NSCLC cell lines, mir-200ba429 was methylated in 13 (38%), partially methylated in 8 (24%), and unmethylated in 13 (38%) cell lines. MiR-200c141 was methylated in 10 (29%), partially methylated in 8 (24%), and unmethylated in 16 (47%) cell lines. We found that the expression of miR-200s was silenced in most of the methylated or partially methylated cell lines. We treated 6 cell lines (3 unmethylated and 3 methylated) with the DNA-demethylating agent 5-aza-2-deoxycytidine (5-Aza) and found that the miR-200s expression levels were restored after treatment with 5-Aza in miR-200s-methylated cells (Supplementary Fig. S1), suggesting that the miR-200s expression levels were regulated by promoter methylation.

### Functional analysis of miR-200s in NSCLC cells

**Figure 2:**
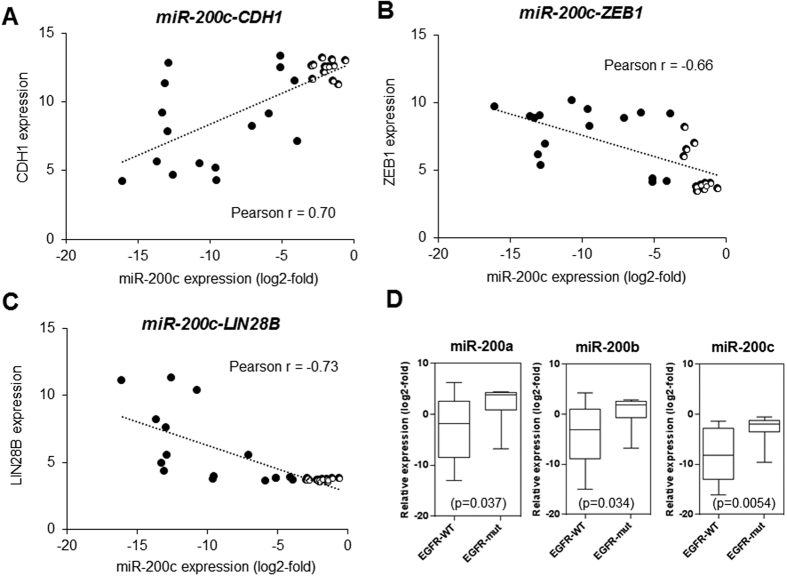
Functional analysis of miR-200s in NSCLC cells. (**A**) Correlation between the miR-200c expression level and CDH1 expression in 28 NSCLC cell lines. Each circle represents NSCLC cell lines. Closed circle, miR-200c low expression group; Opened circle, miR-200c high expression group. (**B**) Correlation between the miR-200c expression level and ZEB1 expression in 28 NSCLC cell lines. (**C**) Correlation between the miR-200c expression level and LIN28B expression in 28 NSCLC cell lines. (**D**) Correlation between EGFR-mutation statuses and miR-200s expressions in 34 NSCLC cell lines. WT, wild type; mut, mutation.

To define the functional significance of genes regulated by the miR-200c status, we performed a gene set enrichment analysis (GSEA) using the Cancer Cell Line Encyclopedia (CCLE) database. Among 34 NSCLC cell lines, comprehensive gene expression data for 28 cell lines were available in the CCLE database, and these cell lines were categorized into an miR-200c-low group (n =** **16) and an miR-200c-high group (n** **=** **12) based on the miR-200c expression and methylation status (Supplementary Table S1). We found that several pathways, including genes defining an EMT (HALLMARK_EPITHELIAL_MESENCHYMAL_TRANSITION), and a subgroup of genes regulated by Myc (HALLMARK_MYC_TARGETS_V1), were positively enriched in the miR-200c-low group (Table 1). We then comprehensively analyzed the correlation between miR-200c expression and the gene expression status (Supplementary Table S2). The epithelial marker *CDH1* was positively correlated with miR-200c expressions (Pearson r** **=** **0.70, *P*** **<** **0.0001) (Fig. 2A), and the mesenchymal marker *ZEB1* was negatively correlated (Pearson r** **=** **−0.66, *P*** **<** **0.0001) (Fig. 2B). Interestingly, *LIN28B*, an oncogenic stem-cell factor^27^, showed a negative correlation with miR-200c expression to the same extent as the correlation between *CDH1* or *ZEB1* and miR-200c (Pearson r** **=** **−0.73, p** **<** **0.0001) (Fig. 2C). In western blots of 34 NSCLC cell lines and HBEC-5KT, miR-200c-silenced NSCLC cells exhibited a low E-cadherin expression level and high vimentin or ZEB1 expression levels (Supplementary Fig. S2). We also investigated the correlation between miR-200s and EMT markers in breast cancer, colon cancer and gastric cancer cell lines. As shown in Supplementary Fig. S3, in all three carcinomas, the expression of miR-200s was low in the cell lines harboring EMT features. These findings are presumed to supplement the relevance of this miR-200c expression based classification and importance of LIN28B.

### Associations between miR-200c statuses and oncogenic alterations

Among the 34 NSCLC cell lines that were examined, 10 cell lines harbored an *EGFR* mutation (*EGFR*-mut), 7 cell lines harbored a *KRAS* mutation (*KRAS*-mut), 4 cell lines harbored a *BRAF* mutation (*BRAF*-mut), 1 cell line harbored a *HER2* mutation (*HER2*-mut), 1 cell line harbored an *NRAS* mutation (*NRAS*-mut), and 11 cell lines harbored none of these mutations (Supplementary Table S1). In 10 cell lines with *EGFR*-mut, miR-200ba429 and miR-200c141 were unmethylated in 8 cell lines and partially methylated in 2 cell lines (HCC2279 and H1650) (Table S1). In addition, the miR-200s expression levels were significantly higher in the *EGFR*-mut cell lines than in the *EGFR* wild-type cell lines (Fig. 2D; *P*** **=** **0.037 in miR-200a, *P*** **=** **0.034 in miR-200b, and *P*** **=** **0.0054 in miR-200c). The similar tendency was observed in 28 NSCLC cell lines which were examined by GSEA (Supplementary Table S3; *P*** **=** **0.044).

### MiR-200c introduction showed an antitumor effect in cells with acquired EGFR-TKI resistance

**Figure 3:**
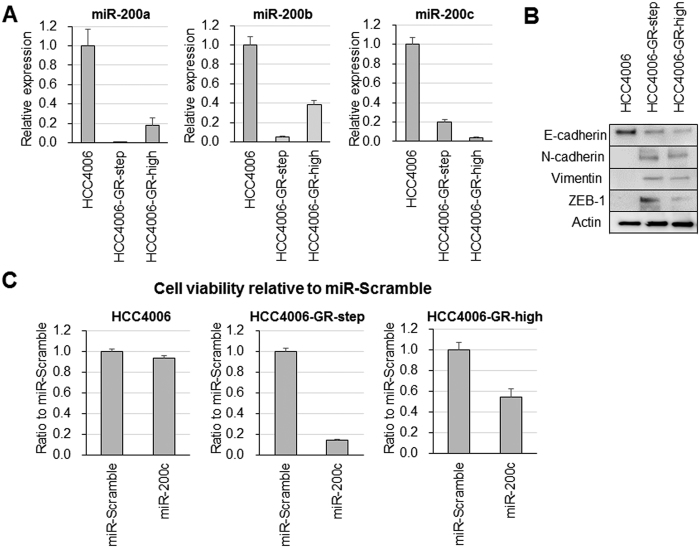
Antitumor effect of miR-200c introduction in parental and acquired EGFR-TKI resistance cells. (**A**) Relative miR-200c expression level using qRT-PCR in parental HCC4006 cells and acquired EGFR-TKI-resistant HCC4006-GR cells. (**B**) EMT-related protein expression level using western blotting in HCC4006 and HCC4006-GR cells. The blots of whole membrane are presented in Supplementary Fig. S5. (**C**) Cell viability after miR-200c transfection in HCC4006 and HCC4006-GR cells using MTT assay.

As we previously reported, EMT features are correlated with the acquisition of EGFR-TKI resistance, but the mechanisms responsible for EGFR-TKI resistance remain unclear. We utilized experimentally established cells with acquired EGFR-TKI resistance and EMT features. By continuously exposing the cells to gefitinib, we established the EGFR-TKI-resistant clones HCC4006-GR-step and HCC4006-GR-high from the *EGFR*-mut HCC4006 cell line^15^. The established cell line showed EMT features, while lacking both T790M secondary *EGFR*-mut and *MET*-amplification. The IC values against gefitinib determined using an MTS assay was 0.024 μmol/L for HCC4006 and over 10 μmol/L for HCC4006-GR cells. MiR-200s expression was suppressed in HCC4006-GR cells compared with the parental HCC4006 (Fig. 3A). In western blots, the HCC4006-GR cells showed EMT features with the downregulation of E-cadherin and the upregulation of N-cadherin, vimentin, and ZEB-1 compared with HCC4006 (Fig. 3B). We examined the effect of miR-200s introduction against cell viability in HCC4006 and HCC4006-GR-high. Although the cell viability of HCC4006 was not affected by miR-200c introduction, the cell viability of HCC4006-GR cells was highly suppressed by miR-200c introduction (Fig. 3C and Supplementary Fig. S3).

### MiR-200c introduction suppressed LIN28B expression and produced an antitumor effect in EGFR-TKI resistant cells

**Figure 4:**
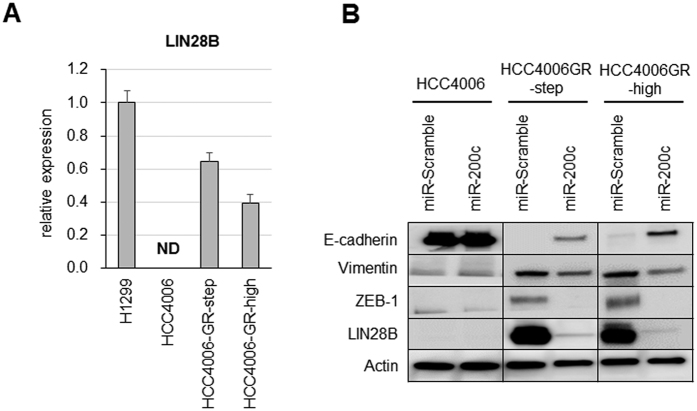
Forced miR-200c expression leads to suppression of LIN28B expression. (**A**) LIN28 expression level as determined using qRT-PCR in HCC4006 and HCC4006-GR cells. ND, not-determined. The LIN28B expression level in H1299 was set at 1, and the relative expression levels in HCC4006 parental and resistance cell lines were shown. (**B**) EMT-related proteins and LIN28B expression level using western blotting after pre-miR-200c or miR-Scramble transfection in HCC4006 and HCC4006-GR cells. The blots of whole membrane are presented in Supplementary Fig. S5.

From the database analysis, miR-200c silencing was found to be correlated with the upregulation of *LIN28B*. Indeed, *LIN28B* was upregulated in HCC4006-GR cells, compared with that in HCC4006, as determined using qRT-PCR (Fig. 4A). When we introduced miR-200c into HCC4006 and HCC4006-GR cells, the expressions of not only mesenchymal protein ZEB1, but also LIN28B were significantly suppressed. On the other hand, expression of E-cadherin, which is known as an epithelial marker, was induced in HCC4006-GR cells (Fig. 4B).

### Knockdown of LIN28B suppressed cell viability in cells with acquired EGFR-TKI resistance

**Figure 5:**
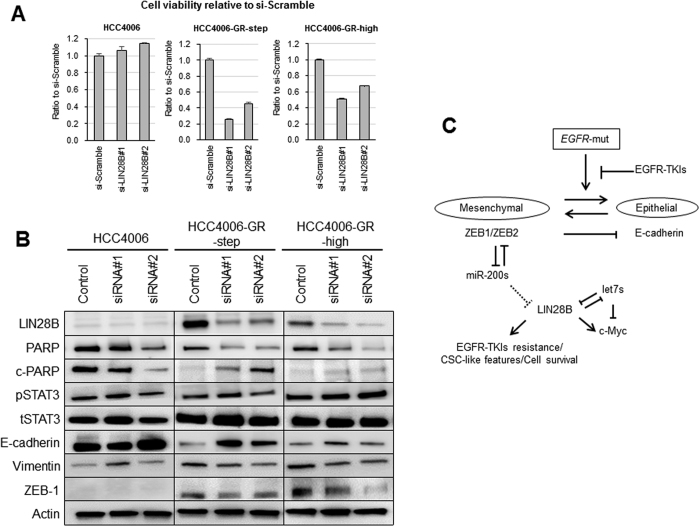
Antitumor effect of LIN28B knockdown in parental and acquired EGFR-TKI resistance cells. (**A**) Cell viability after LIN28B knockdown in HCC4006 and HCC4006-GR cells as determined using an MTT assay. (**B**) Expression of the apoptosis marker c-PARP and EMT marker after LIN28B knockdown as determined using western blotting. The blots of whole membrane are presented in Supplementary Fig. S5. (**C**) Diagram of the mechanism of the EGFR-TKI-mediated miR-200s/LIN28B interaction causing an EMT and acquired EGFR-TKI resistance in EGFR-mut NSCLC cells.

To examine the role of LIN28B on cell survival in cells with acquired EGFR-TKI resistance and EMT features, we suppressed the expression of LIN28B using two kinds of si-RNA. Interestingly, LIN28B suppression using siRNA produced an antitumor effect in HCC4006-GR cells, whereas it was not effective in HCC4006 cells when evaluated using the tetrazolium salt, 3-4,5 dimethylthiazol-2,5 diphenyl tetrazolium bromide (MTT) (Fig. 5A). In a western blot analysis, the knockdown of LIN28B led to the expression of the apoptosis marker c-PARP in HCC4006-GR cells (Fig. 5B). Regarding EMT features, In HCC4006-GR cells, even though the difference is not so clear, the knockdown of LIN28B seems to induce the E-cadherin expression and to reduce the vimentin and ZEB1 expression. This result suggests the possibility that LIN28B is one of the mediator of epithelial to mesenchymal transition in TKI resistance cell line. In addition, we examined phosphorylation of STAT3 as an indicator of TGF-beta/IL6 pathway. As a result, the knockdown of LIN28B doesn’t seem to affect the phosphorylation of STAT3, suggesting that LIN28B doesn’t regulate TGF-beta/IL6 pathway.

### MiR-200c downregulation and LIN28B upregulation after EGFR-TKI resistance acquisition on NSCLC tissues

**Figure 6:**
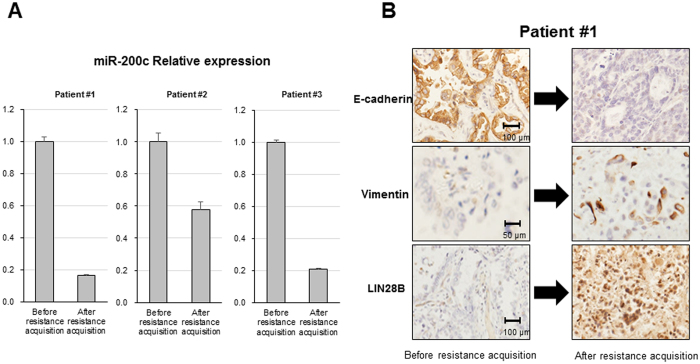
MiR-200c downregulation and LIN28B upregulation after EGFR-TKI resistance acquisition on NSCLC tissues. (**A**) Mir-200c expression level as determined using qRT-PCR in NSCLC patients. (**B**) E-cadherin, vimentin and LIN28B expression level as determined by immunohistochemistry. The images of a representative patient are shown.

We randomly selected the 3 non-small cell lung cancer patients who were treated at our hospital. All 3 patients underwent pulmonary resection as an initial treatment. After relapse, they were treated with EGFR-TKI and acquired resistance finally. We investigated the alteration of EMT marker and the expression of LIN28B and miR-200c before and after EGFR-TKI treatment. The alteration of e-cadherin, vimentin and LIN28B was analyzed by immunohistochemistry and that of miR-200c was analyzed by qRT-PCR. As a result, we confirmed that the expression of miR-200c was significantly suppressed, compared to its expression before resistance acquisition in all three patients (Fig. 6A). On the other hand, LIN28B was highly expressed, compared to before resistance acquisition (Fig. 6B). And then, the expression of E-cadherin was reduced and that of vimentin was increased, indicating that tumors acquired the EMT feature in the process of EGFR-TKI resistance acquisition (Fig. 6B). These results were consistent with those of HCC4006 parental and GR cells.

## Discussion

In this study, we found that the miR-200c/LIN28B axis plays a critical role in the cell viability of acquired EGFR-TKI resistance cells harboring EMT features. Acquired resistance cells showed miR-200s silencing and LIN28B overexpression, which was the same for clinical samples. MiR-200c introduction caused LIN28B suppression, and both miR-200c introduction and LIN28B suppression had an anti-proliferative effect in HCC4006-GR cells. These effects were not observed in parental HCC4006 cells lacking miR-200s silencing and LIN28B overexpression, suggesting that the survival of acquired resistance cells depends on the miR-200c/LIN28B axis (Fig. 5C). It is still inconclusive that miR-200c directly or indirectly regulates LIN28B. Therefore, further investigation is mandatory.

We found that miR-200c expression was not silenced in most of the *EGFR*-mut NSCLC cell lines. Several reports have shown that *EGFR*-mut is associated with epithelial characteristics in NSCLC^28^,^29^. The detailed mechanisms of how *EGFR*-mut leads to the maintenance of epithelial characteristics have been unclear. Takeyama *et al*. hypothesized that *EGFR*-mut cells are resistant to EMT-inducing signals, or that *EGFR*-mut cells specifically express genes that retain cells in the epithelial state^29^. On the other hand, several reports have shown that the chronic activation of EGFR can promote an EMT-like change^30^,^31^. Further investigation is required regarding *EGFR*-mut and epithelial characteristics, including E-cadherin expression, because E-cadherin expression has been proposed as a biomarker predicting the clinical activity of EGFR-TKIs in NSCLC^12^.

We and other groups have reported that EGFR-TKIs cause an EMT change with miR-200s silencing in a subset of NSCLC harboring *EGFR*-mut^14^. EMT is a phenomenon in which cells with epithelial phenotypes acquire mesenchymal characteristics, and EMT plays an important role in cancer metastasis and drug resistance. EMT has been shown to be correlated with a poor prognosis in multiple epithelial-derived solid tumors. In preclinical models and clinical samples, EMT features were observed after the acquisition of resistance to EGFR-TKIs. Although the detailed mechanisms explaining how these alterations cause EGFR-TKIs resistance have been unclear, we have identified LIN28B as a candidate oncogenic driver molecule in acquired EGFR-TKI resistance cells.

LIN28B and its paralog LIN28A are RNA-binding proteins that mediate diverse biological functions. The LIN28 family regulates mammalian stem cell self-renewal, and LIN28A in combination with NANOG, OCT4, and SOX2, can reprogram human somatic cells to become pluripotent stem cells^32^. LIN28A participates in these key biological processes by blocking the maturation of the tumor suppressor miR let-7 family^33^,^34^. Furthermore, LIN28A plays a functional role in the maintenance of the ALDH1-positive CSC population in tumors^35^. LIN28B also influences multiple oncogenic signaling networks in diverse cellular contexts, and is being recognized as oncogenic stem-cell factor^27^,^36^. Indeed, CD166-positive tumor-initiating cells obtained from primary NSCLC tumor expressed high levels of LIN28B^27^. In addition, LIN28B has been reported as a candidate biomarkers of resistance to platinum-based chemotherapy and of a poor clinical outcome in patients with epithelial ovarian carcinoma^37^, suggesting the function of LIN28B as a drug-resistant factor.

LIN28B expression is reportedly induced by the c-Myc oncogenic transcription factor in multiple human and mouse tumor models^38^. The upregulation of LIN28 family and Myc or the downregulation of let-7 may promote the conversion of epithelial cells to a more undifferentiated stage and maintain the tumor cells in this stem-like stage^35^,^38^. In our analysis, genes regulated by Myc were positively enriched in the miR-200c-low group when examined using GESA (Table 1). These findings are compatible with the result that miR-200c-silenced cells had a high level of LIN28B expression as well as EMT features.

MiR-200c-introduced cells showed the downregulation of LIN28B in NSCLC cells, and miR-200c introduction produced an anti-proliferative effect in HCC4006-GR cells. In addition, both miR-200c introduction and LIN28B downregulation inhibit the expression of EMT markers. In prostate cancer, Kong *et al*. also reported that miR-200b and miR-200c introduction downregulated the LIN28B protein^39^. Although the exact mechanism involving the miR-200s and LIN28B interaction remains unclear, it is possible that both the miR-200s and the let-7 miR families are composed of multiple members, with each member regulating largely overlapping sets of targets^40^.

In conclusion, the miR-200c/LIN28B axis plays an important role in acquired resistance to EGFR-TKI and might be a useful therapeutic target for overcoming EMT-related EGFR-TKI resistance. To date, there are no drugs directly targeting miR-200c or LIN28B expression. Further investigation of the modulation of the miR-200c/LIN28B axis may lead to further successes in EGFR-TKIs treatment.

## Materials and Methods

### Ethics statement

This study was approved by the Institutional Review Board/Ethical Committee of Okayama University and all experiments were performed in accordance with relevant guidelines and regulations.

### Cell lines and reagents

We used 34 human NSCLC cell lines, 4 breast cancer cell lines, 3 colon cancer cell lines, 4 gastric cancer cell lines, 1 bronchial epithelial cell line (HBEC-5KT), and 1 embryonic kidney cell line (HEK293T). The details of 34 NSCLC cell lines and 1 bronchial epithelial cell line are shown in Supplementary Table S1. All cell lines except for PC-9 and A549 were kindly provided by Dr. Adi F. Gazdar (Hamon Center for Therapeutic Oncology Research and Department of Pathology, University of Texas Southwestern Medical Center, Dallas, TX). PC-9 was obtained from Immuno-Biological Laboratories (Takasaki, Gunma, Japan). A549 was obtained from the American Type Culture Collection (Manassas, VA). All the NSCLC cell lines except for H3255 were maintained in RPMI-1640 (Thermo Fisher Scientific, Waltham, MA) supplemented with 10% fetal bovine serum (FBS). H3255 was maintained in ACL-4. HEK293T cells were cultured in Dulbecco’s Modified Eagle Medium supplemented with 10% FBS. HBEC5-KT was maintained in Keratinocyte-SFM medium with bovine pituitary extract and human recombinant epidermal growth factor (all from Thermo Fisher Scientific)^41^. In addition, we used an experimentally established EGFR-TKI resistant cell line with an *EGFR* activation mutations (19del), HCC4006-GR-step and HCC4006-GR-high, as previously reported^15^. The identities of all the cell lines were confirmed by analyzing the short tandem repeat profile using the Cell ID System (Promega, Madison, WI) and an ABI Prism 310 Genetic Analyzer (Thermo Fisher Scientific), according to the manufacturer’s instructions. The EGFR-TKI gefitinib was purchased from InvivoGen (San Diego, CA).

### DNA, RNA and miRNA extraction

Genomic DNAs were extracted from cell lines using a DNeasy Blood and Tissue Kit (Qiagen, Venlo, Netherlands). Total RNAs were extracted from cell lines using an RNeasy Mini Kit (Qiagen). Complementary DNA (cDNA) was synthesized from the total RNA using High-Capacity cDNA Reverse Transcription Kits (Thermo Fisher Scientific), according to the manufacturer’s instructions. The miRNA was isolate from cell lines with TaqMan MicroRNA Cells-to-CT Kit (Ambion) and total RNA was extracted from microdissected formalin-fixed, paraffin-embedded (FFPE) tissues by using a miRNeasy FFPE Kit (Qiagen). Reverse transcription was conducted with TaqMan Micro-RNA Reverse Transcriptional Kit systems (Applied Biosystems).

### DNA direct sequencing and methylation analysis

We determined the mutational status of *EGFR, HER2, KRAS, NRAS*, and *BRAF* genes in NSCLC cell lines using direct sequencing, as previously reported^15^. In a methylation analysis, DNA was subjected to bisulfate treatment using the Epitect Bisulfite Kit (Qiagen), according to the manufacturer’s protocol. The DNA methylation statuses of miR-200ba429 and miR-200c141 were examined using MSP, as previously reported (Supplementary Table S4A)^15^. To restore the gene expression that was reduced by methylation, cells were treated with 5-Aza (Sigma-Aldrich, St. Louis, MO) at a concentration of 5 μmol/L for 6 days.

### mRNA and miRNA expression analysis using quantitative reverse transcription-PCR

Gene expression was analyzed using qRT-PCR and cDNAs, TaqMan Gene Expression Assays, and the ABI StepOnePlus Real-Time PCR Instrument (Thermo Fisher Scientific). The expressions of mRNA and miR were calculated using the delta-delta-CT method. The glyceraldehyde-3-phosphate dehydrogenase gene and miR-374 were used as endogenous controls for the mRNA and miR expression analyses, respectively. The primer and probe sets were purchased from Thermo Fisher Scientific and were used according to the manufacturer’s instructions (Table S4B).

### Gene expression analysis in CCLE cell line panel and functional analysis in GSEA

Comprehensive gene expression data for 28 NSCLC cell lines were obtained from the CCLE (http://www.broadinstitute.org/ccle/)^42^. Twenty-eight NSCLC cell lines were categorized into a miR-200c low group (n** **=** **16) or a miR-200c high group (n** **=** **12) based on the miR-200c expression level and methylation status, and functional analyses of the NSCLC cell lines were performed using GSEA (Molecular Signatures Database v5.0)^43^.

### Western blotting analysis

The detailed protocol for western blotting has been described previously^44^. The primary antibodies were as follows: anti-E-cadherin, N-cadherin, EGFR, EGFR-19del (exon19 E746-A750del specific), vimentin (R28), LIN28B (Cell Signaling Technology, Beverly, MA), and ZEB1 (H-102, Santa Cruz Biotechnology, Santa Cruz, CA). Monoclonal anti-actin antibody, used as an equal loading control, was purchased from Merck Millipore (Billerica, MA). The following secondary antibodies were used: goat anti-rabbit or anti-mouse immunoglobulin G (IgG)-conjugated horseradish peroxidase (Santa Cruz Biotechnology). To detect specific signals, the membranes were examined using ECL Prime Western Blotting Detection System (GE Healthcare, Amersham, UK).

### Pre-miRNA transfection

NSCLC cells were transfected with 50 nmol/L of pre–miR-200c (has-miR-200c-3p, #MC11714) or control-miR (miR-Scrambled) using Lipofectamine RNAiMAX (all from Thermo Fisher Scientific), total amount of miR-mimics was 300 pmol, once a day for 2 days. The cells were used in the assays at 48 hours after the final transfection.

### SiRNA transfection

NSCLC cells were transfected with 5 nmol/L of Silencer Select siRNA against *LIN28B* (si-LIN28B#1 and si-LIN28B#2) or scrambled negative control siRNA (si-Scramble) (Thermo Fisher Scientific) by using Lipofectamine RNAiMAX and were incubated for 48 hours.

### Determination of cell proliferation

The IC50 value against gefitinib was determined using a modified MTS assay with CellTiter 96 AQueous One Solution Reagent (Promega) as previously reported^15^. For experiments testing the effect of miR-200c transfection or the knockdown of siRNA on cell proliferation, the MTT (Sigma-Aldrich, St. Louis, MO) dye reduction method was used. Cells were cultured at 37 °C with 5% CO_2_, in 6-well plates at a concentration of 1** **×** **10^5^ cells/mL for 72–96 h. MTT was dissolved in RPMI-1640, and 100 μL of the MTT solution were added to each well; the plates were then incubated at 37 °C with 5% CO_2_ for 2 h. Subsequently, 100 μL of dimethyl sulfoxide were added to each well. The cell viability was assessed by measuring the optical density at 570 nm and 690 nm on a plate reader. Three independent experiments consisting of triplicate runs (at least) were performed.

### Immunohistochemical analysis of clinical samples

Lung cancer tissues were obtained from the patients who underwent surgery at Okayama University Hospital (Okayama, Japan). This experimental protocol was approved by the Institutional Review Board/Ethical Committee of Okayama University (No. 147) and informed consent were obtained from all the patients. Tissue samples were fixed in 10% formaldehyde and embedded in paraffin.

Immunohistochemical (IHC) staining with E-cadherin (diluted 1:1000 in PBS), vimentin (1:200), LIN28B (1:100) was conducted. The detailed protocol for the IHC staining has been described previously^45^.

### Statistical analyses

All the statistical analyses were performed using GraphPad Prism 6 (GraphPad Software). P** **<** **0.05 was considered statistically significant. All the tests were two-sided.

## Additional Information

**How to cite this article**: Sato, H. *et al*. Targeting the miR-200c/LIN28B axis in acquired EGFR-TKI resistance non-small cell lung cancer cells harboring EMT features. *Sci. Rep.* **7**, 40847; doi: 10.1038/srep40847 (2017).

**Publisher's note:** Springer Nature remains neutral with regard to jurisdictional claims in published maps and institutional affiliations.

## Supplementary Material

Supplementary Information

## Figures and Tables

**Table 1:**
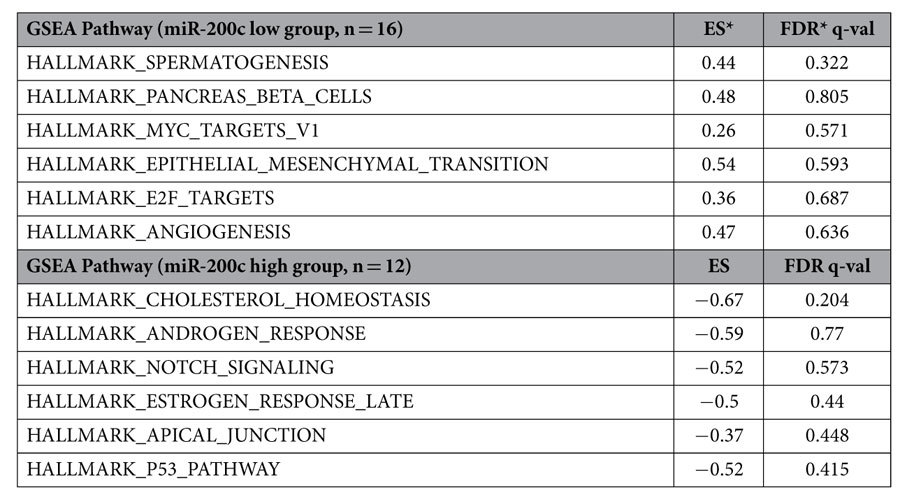
Enriched pathways in the cells with miR-200c-low as well as miR-200c-high expression.

ES: enriched score.

FDR: False discovery rate.
