# Supplementary material for: Targeting the miR-200c/LIN28B axis in acquired EGFR-TKI resistance non-small cell lung cancer cells harboring EMT features
Source: Sci Rep. 2017 Jan 13;7:40847. doi: 10.1038/srep40847 (PMC5233972; doi:10.1038/srep40847)

Title

Targeting the miR-200c/LIN28B axis in acquired EGFR-TKI resistance non-small cell lung cancer cells harboring EMT features

Authors

Hiroki Sato[1+], Kazuhiko Shien[1+], Shuta Tomida[2], Kazuhiro Okayasu[1], Ken Suzawa[1], Shinsuke Hashida[1], Hidejiro Torigoe[1], Mototsugu Watanabe[1], Hiromasa Yamamoto[1], Junichi Soh[1], Hiroaki Asano[1], Kazunori Tsukuda[1], Shinichiro Miyoshi[1], Shinichi Toyooka[1,3*]

Affiliation

Departments of [1]General Thoracic Surgery and Breast and Endocrinological Surgery, [2]Bioinformatics, [3]Clinical Genomic Medicine, Okayama University Graduate School of Medicine, Dentistry and Pharmaceutical Sciences, Okayama, Japan

Supplementary Figure legends

Supplementary Figure S1

Changes in miR-200s expressions after 5-Aza treatment in miR-200s methylated (upper row) and unmethylated (lower row) NSCLC cells as examined using quantitative reverse transcription-PCR (qRT-PCR).

Supplementary Figure S2

The EMT-related protein expression level was examined using western blotting. MiR200c-silenced and methylated (left side) cells showed the suppression of E-cadherin and the upregulation of vimentin and ZEB-1.

Supplementary Figure S3

The correlation between miR-200s and EMT markers in breast cancer, colon cancer and gastric cancer cell lines. The miR-200a, miR-200b, and miR-200c expression statuses as determined using qRT-PCR. The miR-200s expression levels in HBEC-5KT were set at 1, and the expression levels in the breast, colon and gastric cell lines were shown relative to those in the HBEC-5KT cell line. EMT-related proteins expression level as determined using western blotting.

Supplementary Figure S4

Relative miR-200c expression level using RT-PCR in HCC4006 and HCC4006-GR cells after pre-miR-200c or scrambled control (miR-Scramble) transfection. We confirmed the mir-200C enough introduction.

Supplementary Figure S5

The blots of whole membrane in the main document are presented.

Supplementary Table S1. Characteristics of NSCLC cell lines

Cell lines	Histology	Genetic alterations	miR-200c141 methylation	miR-200c exp/HBEC5KT	miR-200c high/low group
H1299	LC	*NRAS* mut	M	0.000015	Low
H125	AdSq	WT	M	0.000021	ND
H157	Sq	*KRAS* mut	M	0.000054	ND
H522	Ad	WT	M	0.000080	Low
H23	Ad	*KRAS* mut	M	0.00010	Low
H2087	Ad	*BRAF* mut	M	0.00012	Low
A549	Ad	*KRAS* mut	M	0.00013	Low
H2009	Ad	*KRAS* mut	M	0.00014	Low
H661	LC	WT	M	0.00017	Low
H838	Ad	WT	M	0.00060	Low
H1819	Ad	WT	M/U	0.0011	ND
HCC2279	Ad	*EGFR* mut	M/U	0.0013	Low
H460	Ad	*KRAS* mut	M/U	0.0014	Low
HCC366	AdSq	WT	M/U	0.0077	Low
HCC364	Ad	*BRAF* mut	M/U	0.017	Low
H1650	Ad	*EGFR* mut	M/U	0.030	Low
H358	Ad	*KRAS* mut	U	0.030	Low
H1395	Ad	*BRAF* mut	M/U	0.059	Low
H2228	Ad	WT	M/U	0.069	Low
H820	Ad	*EGFR* mut	U	0.12	ND
Calu-3	Ad	WT	U	0.13	High
H1975	Ad	*EGFR* mut	U	0.14	High
H441	Ad	*KRAS* mut	U	0.15	High
HCC827	Ad	*EGFR* mut	U	0.22	High
H2170	Sq	WT	U	0.24	High
H1781	Ad	*HER2* mut	U	0.25	High
H1993	Ad	WT	U	0.26	ND
HCC4006	Ad	*EGFR* mut	U	0.30	High
H3255	Ad	*EGFR* mut	U	0.36	High
H1666	Ad	*BRAF* mut	U	0.38	High
HCC4011	Ad	*EGFR* mut	U	0.38	ND
H1648	Ad	WT	U	0.38	High
PC-9	Ad	*EGFR* mut	U	0.47	High
HCC2935	Ad	*EGFR* mut	U	0.67	High
HBEC-5KT	NBC	WT	U	1	-

H (in cell line name), NCI-H; LC, large cell carcinoma; AdSq, adeno-squamous cell carcinoma; Ad, adenocarcinoma; Sq, squamous cell carcinoma;; NBC, normal bronchial cell; mut, mutation; WT, wild type; M, methylated; M/U, partially methylated; U, unmethylated, ND, no data in CCLE database

Supplementary Table S2

Correlation between miR-200c expression and gene expression in NSCLC cell

Rank	gene_ID	probe_ID	pearson r	*p* value
1	NA	238884_at	0.793720749	4.663E-07
2	CYB561	209163_at	0.779312746	1.027E-06
3	ESRP2	229223_at	0.776797827	1.171E-06
4	ATP8B1	226302_at	0.775038026	1.283E-06
5	NA	236279_at	0.773739066	1.372E-06
6	GALNT3	203397_s_at	0.772365921	1.472E-06
7	NA	228441_s_at	0.772038015	1.496E-06
8	SPINT1	202826_at	0.766909056	1.936E-06
9	C6orf132	238028_at	0.763373391	2.303E-06
10	TAPBPL	218747_s_at	0.762097306	2.450E-06
11	KIAA0247	202181_at	0.758013583	2.981E-06
12	NA	228440_at	0.755021123	3.433E-06
13	EHF	225645_at	0.752184163	3.917E-06
14	LGALS3BP	200923_at	0.747485799	4.855E-06
15	PKP3	209873_s_at	0.746974723	4.969E-06
16	CDS1	205709_s_at	0.746612297	5.051E-06
17	MAL2	224650_at	0.745714746	5.259E-06
18	IRF6	202597_at	0.743950216	5.690E-06
19	KIAA1522	224746_at	0.743238984	5.873E-06
20	MPZL2	203780_at	0.742917991	5.957E-06
21	RASEF	235144_at	0.74210094	6.176E-06
22	C1orf116	219476_at	0.741270166	6.406E-06
23	SH2D3A	219513_s_at	0.739670058	6.872E-06
24	ESRP2	219395_at	0.736729845	7.806E-06
25	CLDN4	201428_at	0.735742444	8.145E-06
26	ELF3	210827_s_at	0.735402339	8.265E-06
27	NFKBIZ	223218_s_at	0.734327179	8.653E-06
28	RAB27B	228708_at	0.733640326	8.910E-06
29	DENND1B	228032_s_at	0.73238208	9.398E-06
30	SCNN1A	203453_at	0.732011488	9.546E-06
31	MYO5B	225299_at	0.730586707	1.014E-05
32	TAPBPL	218746_at	0.729940099	1.041E-05

33	CDS1	226185_at	0.725998121	1.226E-05
34	KRT19	201650_at	0.72472124	1.292E-05
35	LAD1	216641_s_at	0.722922447	1.391E-05
36	DENND1B	219696_at	0.722298117	1.426E-05
37	EPS15L1	231926_at	0.720901223	1.509E-05
38	EHF	232361_s_at	0.72019736	1.552E-05
39	SMARCA4	212520_s_at	0.719484976	1.597E-05
40	CYB561	209164_s_at	0.719242806	1.613E-05
41	KIAA1217	231807_at	0.71840833	1.667E-05
42	NA	236616_at	0.718349706	1.671E-05
43	LAD1	203287_at	0.716083937	1.828E-05
44	DENND1B	1564164_at	0.715856787	1.845E-05
45	FXYD3	202489_s_at	0.714766401	1.926E-05
46	PPA2	1559496_at	0.714000317	1.984E-05
47	SMARCA4	208794_s_at	0.713772255	2.002E-05
48	ARHGEF5	204765_at	0.713071077	2.058E-05
49	SMARCA4	213720_s_at	0.712167237	2.131E-05
50	MSMO1	209146_at	0.711938062	2.150E-05
51	MPZL3	1570585_at	0.711686159	2.171E-05
52	EPS8L2	218180_s_at	0.710931179	2.236E-05
53	NA	235924_at	0.70959819	2.353E-05
54	IRF6	1552477_a_at	0.709547512	2.358E-05
55	NFKBIZ	223217_s_at	0.70948421	2.364E-05
56	TMEM184A	1558281_a_at	0.709119493	2.397E-05
57	LGALS3	208949_s_at	0.709046286	2.404E-05
58	EHF	232360_at	0.707711274	2.529E-05
59	MAPK13	210059_s_at	0.707135654	2.585E-05
60	SERINC5	212812_at	0.705133929	2.789E-05
61	CTAGE5	204055_s_at	0.704688287	2.836E-05
62	HOOK2	218780_at	0.70448851	2.858E-05
63	B3GNT3	204856_at	0.701839392	3.155E-05
64	HS3ST1	205466_s_at	0.701266228	3.223E-05
65	CDH1*	201131_s_at	0.699566608	3.432E-05
66	PRRG4	238513_at	0.699438444	3.449E-05
67	MARVELD3	239148_at	0.699435198	3.449E-05

68	INADL	223681_s_at	0.698956497	3.510E-05
69	PRSS22	205847_at	0.698922938	3.515E-05
70	KRT7	209016_s_at	0.698879508	3.520E-05
71	SH3YL1	204019_s_at	0.697357689	3.722E-05
72	CDS1	226187_at	0.696709828	3.811E-05
73	TNFRSF21	214581_x_at	0.696627051	3.823E-05
74	ST14	216905_s_at	0.695989722	3.913E-05
75	LAMB3	209270_at	0.695764219	3.945E-05
76	NA	238742_x_at	0.695730092	3.950E-05
77	RASEF	1553986_at	0.694785976	4.087E-05
78	ELMO3	219411_at	0.694735173	4.095E-05
79	NA	242598_at	0.694667756	4.105E-05
80	EHF	224189_x_at	0.69344282	4.290E-05
81	C1orf116	228865_at	0.693359167	4.303E-05
82	CLDN7	202790_at	0.693218306	4.325E-05
83	ITGB4	204990_s_at	0.693166734	4.333E-05
84	SMARCA4	214728_x_at	0.691845352	4.543E-05
85	EPN3	223895_s_at	0.691273238	4.637E-05
86	CC2D1A	58994_at	0.691147138	4.658E-05
87	DAAM1	226666_at	0.69091078	4.697E-05
88	GPR56	212070_at	0.69081241	4.714E-05
89	RNF144B	228153_at	0.69003879	4.845E-05
90	MPZL3	227747_at	0.689961781	4.859E-05
91	ESRP1	225846_at	0.689570112	4.927E-05
92	SHANK2	213308_at	0.689538464	4.932E-05
93	EPCAM	201839_s_at	0.689227229	4.987E-05
94	NA	226725_at	0.68852021	5.113E-05
95	MAPK13	210058_at	0.688457066	5.124E-05
96	ZHX2	203556_at	0.687905156	5.225E-05
97	C11orf52	238805_at	0.687853482	5.235E-05
98	NA	1565598_at	0.687451079	5.309E-05
99	LGALS8	208933_s_at	0.687144883	5.367E-05
100	SRCAP	38766_at	0.687043206	5.386E-05
101	ELF3	201510_at	0.686660968	5.459E-05
102	SLFN5	238430_x_at	0.686291752	5.530E-05

103	DDR1	207169_x_at	0.685672012	5.651E-05
104	EPN3	220318_at	0.685480021	5.689E-05
105	DDR1	1007_s_at	0.68495064	5.795E-05
106	TMC4	226403_at	0.68461484	5.863E-05
107	AGRN	212285_s_at	0.684556154	5.875E-05
108	LRG1	228648_at	0.684512446	5.884E-05
109	TC2N	1553132_a_at	0.684253527	5.937E-05
110	TNKS1BP1	224792_at	0.683828639	6.025E-05
111	SYTL1	227134_at	0.683523204	6.089E-05
112	TACSTD2	202286_s_at	0.680564328	6.742E-05
113	DDR1	208779_x_at	0.68039145	6.782E-05
114	MACC1	232151_at	0.679431811	7.008E-05
115	NA	1565863_at	0.678777862	7.166E-05
116	KIAA0284	213242_x_at	0.678532162	7.226E-05
117	MYO5B	225301_s_at	0.678414821	7.255E-05
118	DDR1	210749_x_at	0.678203343	7.307E-05
119	MICALL2	219332_at	0.677923072	7.377E-05
120	SRCAP	213667_at	0.677426754	7.502E-05
121	MPZL2	230518_at	0.675128802	8.106E-05
122	CD46	208783_s_at	0.674956395	8.153E-05
123	MBNL3	229498_at	0.674821537	8.190E-05
124	GRHL2	219388_at	0.674818571	8.191E-05
125	TMEM154	238063_at	0.674511661	8.275E-05
54490	ZEB1*	212764_at	-0.656284285	1.492E-04
54491	NA	1570503_at	-0.656967624	1.461E-04
54492	ELOVL2	213712_at	-0.65704331	1.457E-04
54493	GABRD	208457_at	-0.657463028	1.438E-04
54494	GCFC2	216305_s_at	-0.657911502	1.418E-04
54495	CCDC88A	239233_at	-0.659155688	1.364E-04
54496	NA	213484_at	-0.659769162	1.337E-04
54497	NA	243707_at	-0.66032513	1.314E-04
54498	VRK3	1566701_at	-0.661517598	1.265E-04
54499	SCN3B	204722_at	-0.661681853	1.258E-04
54500	NA	241449_at	-0.662126719	1.241E-04
54501	APBA2	209870_s_at	-0.663185084	1.199E-04

54502	DNAJC18	227166_at	-0.663702148	1.180E-04
54503	RHBDL3	1553268_at	-0.664087207	1.165E-04
54504	JAM3	231721_at	-0.664404925	1.153E-04
54505	DZIP1	204556_s_at	-0.664591237	1.146E-04
54506	NEK3	213116_at	-0.664725768	1.141E-04
54507	RPAP2	222893_s_at	-0.665005604	1.131E-04
54508	SGTB	228745_at	-0.66564759	1.108E-04
54509	TERT	207199_at	-0.665874685	1.100E-04
54510	SENP1	226619_at	-0.666683912	1.071E-04
54511	OSBPL6	238575_at	-0.666939183	1.062E-04
54512	NA	1566482_at	-0.667018055	1.060E-04
54513	UTP18	203721_s_at	-0.667981655	1.027E-04
54514	FNDC3A	238961_s_at	-0.668470686	1.011E-04
54515	TRPV5	1555042_at	-0.66868043	1.004E-04
54516	ELMO2	1554857_at	-0.669568363	9.749E-05
54517	SSX2IP	210871_x_at	-0.669649465	9.723E-05
54518	FGFR1	226705_at	-0.67011199	9.576E-05
54519	PSMD14	212296_at	-0.670458697	9.467E-05
54520	NA	224172_at	-0.670958774	9.312E-05
54521	METTL21A	235177_at	-0.671352439	9.192E-05
54522	NA	1565700_at	-0.671418962	9.172E-05
54523	LHX2	211219_s_at	-0.672712143	8.787E-05
54524	LOC400464	243957_at	-0.673636295	8.521E-05
54525	WDR35	226890_at	-0.674359454	8.317E-05
54526	BVES	228783_at	-0.674403047	8.305E-05
54527	ACOT7	215728_s_at	-0.67461814	8.246E-05
54528	NA	228451_at	-0.674709041	8.221E-05
54529	TYMS	1554696_s_at	-0.675318509	8.054E-05
54530	VPS33A	204590_x_at	-0.675431177	8.024E-05
54531	CC2D2B	219731_at	-0.677449089	7.496E-05
54532	FAM101B	226905_at	-0.677824944	7.402E-05
54533	ACOT7	208002_s_at	-0.678537892	7.225E-05
54534	PGBD1	235411_at	-0.678555396	7.220E-05
54535	NA	220233_at	-0.679099743	7.088E-05
54536	C3orf64	221935_s_at	-0.679406389	7.014E-05

54537	HOXA7	206847_s_at	-0.679836104	6.912E-05
54538	ODF3	233795_at	-0.681093595	6.621E-05
54539	SSX2IP	203016_s_at	-0.682438959	6.322E-05
54540	PRAME	204086_at	-0.68279118	6.245E-05
54541	NA	240136_at	-0.683119546	6.175E-05
54542	NA	237277_at	-0.683347331	6.126E-05
54543	BUB1	209642_at	-0.684014535	5.986E-05
54544	SCN8A	207049_at	-0.684537651	5.879E-05
54545	NA	230604_at	-0.68540677	5.703E-05
54546	LOC339298	1561277_at	-0.685458037	5.693E-05
54547	IGF2BP1	223689_at	-0.688011208	5.206E-05
54548	PCOLCE2	219295_s_at	-0.688080657	5.193E-05
54549	CLEC11A	211709_s_at	-0.688538835	5.110E-05
54550	NA	238471_at	-0.688982285	5.030E-05
54551	C9orf29	1569839_s_at	-0.689923002	4.865E-05
54552	FAM101B	226876_at	-0.690494681	4.767E-05
54553	C3orf15	1554528_at	-0.690495632	4.767E-05
54554	PPAPDC3	224506_s_at	-0.690962414	4.689E-05
54555	MSRB3	1554127_s_at	-0.691547176	4.592E-05
54556	MSRB3	225782_at	-0.694500666	4.130E-05
54557	SLC25A33	223296_at	-0.694637348	4.109E-05
54558	NLRP9	1553527_at	-0.695691655	3.955E-05
54559	LOC100506844	226546_at	-0.696547962	3.834E-05
54560	IGF2BP1	241574_s_at	-0.696807668	3.798E-05
54561	NA	1556749_at	-0.69724066	3.738E-05
54562	MAD2L2	223234_at	-0.6993726	3.457E-05
54563	JAM3	212813_at	-0.699379965	3.456E-05
54564	SSX2IP	203017_s_at	-0.699671784	3.419E-05
54565	OGG1	205760_s_at	-0.700025004	3.375E-05
54566	TAS2R13	221395_at	-0.700554677	3.309E-05
54567	NA	239455_at	-0.702193883	3.114E-05
54568	NA	229373_at	-0.702381195	3.092E-05
54569	BUB1	215509_s_at	-0.703118085	3.008E-05
54570	DYNC1LI1	222479_s_at	-0.707006751	2.598E-05
54571	HOXD9	205604_at	-0.707173192	2.582E-05

54572	LOC285103	1561433_at	-0.708663274	2.439E-05
54573	MSRB3	238583_at	-0.709068009	2.402E-05
54574	EID2B	242470_at	-0.709651034	2.348E-05
54575	KRT2	207908_at	-0.709883178	2.328E-05
54576	HOXC5	206739_at	-0.710757924	2.251E-05
54577	ELOVL2	220029_at	-0.710986574	2.231E-05
54578	ORMDL1	223187_s_at	-0.713739635	2.005E-05
54579	MYT1	1556269_at	-0.716273356	1.815E-05
54580	FHL1	210299_s_at	-0.716692025	1.785E-05
54581	CCDC88A	225045_at	-0.717560204	1.725E-05
54582	FHL1	214505_s_at	-0.718054162	1.691E-05
54583	FHL1	201540_at	-0.718443704	1.665E-05
54584	PPM1E	236302_at	-0.722246456	1.429E-05
54585	DPYSL5	222797_at	-0.72245538	1.417E-05
54586	FHL1	201539_s_at	-0.72328118	1.371E-05
54587	CCT4	200877_at	-0.72351004	1.358E-05
54588	CCDC88A	1562648_at	-0.725067332	1.274E-05
54589	LIN28B*	229349_at	-0.725851515	1.234E-05
54590	FHL1	210298_x_at	-0.726781895	1.187E-05
54591	DKFZp434E1119	1565771_at	-0.728277849	1.116E-05
54592	C17orf104	1562484_at	-0.731641002	9.697E-06
54593	LIX1L	225793_at	-0.733511242	8.959E-06
54594	APBA2	209871_s_at	-0.735375007	8.274E-06
54595	FBXO43	236852_at	-0.735662801	8.173E-06
54596	SSX2IP	203018_s_at	-0.736374794	7.927E-06
54597	SSX2IP	203015_s_at	-0.73679179	7.786E-06
54598	RBM24	235004_at	-0.742591089	6.044E-06
54599	HMGN4	202579_x_at	-0.745037044	5.421E-06
54600	MSRB3	225790_at	-0.745517488	5.305E-06
54601	LOC100505481	230787_at	-0.749959773	4.339E-06
54602	NA	244776_at	-0.752263719	3.902E-06
54603	DLL3	219537_x_at	-0.75600514	3.278E-06
54604	HBQ1	220807_at	-0.757005965	3.126E-06
54605	LIX1L	235036_at	-0.764533496	2.176E-06
54606	ZNF569	1569366_a_at	-0.776153706	1.211E-06

54607	ADD2	230988_at	-0.779072411	1.040E-06
54608	SLC10A4	239913_at	-0.783115245	8.384E-07
54609	ZNF569	1553696_s_at	-0.790010518	5.747E-07
54610	CCDC88A	221078_s_at	-0.801956106	2.887E-07
54611	ADD2	205268_s_at	-0.802499236	2.795E-07
54612	LOC283683	217520_x_at	-0.811479437	1.612E-07
54613	FSD1	219170_at	-0.828766014	5.126E-08

* The status of CDH1, ZEB1 and LIN28B is highlighted in blue.

Supplementary Table S3. Correlation between EGFR-mutation statuses and miR-200s expressions in 28 NSCLC cell lines

		miR200c-Low (n=16)	mir200c-High (n=12)	P value (Fisher's exact test)
EGFR	WT	14	6	
	Mutant	2	6	0.044
All-ras	WT	7	11	
	Mutant	9	1	0.016

Supplementary Table S4

(A) Primers of methylation-specific PCR (MSP)

miRNA		primer sequences (5' to 3')	
**miR-200ba429**	Methylated	Forward	GAGCGGAGATTGGTTAGC
		Reverse	TCGAAAACGACGAAACAATAA
	Unmethylated	Forward	TAGGAGTGGAGATTGGTTAGT
		Reverse	AAATTTCAAAAACAACAAAACAAT
**miR-200c141**	Methylated	Forward	GCGTTGGTTGTTCGGTAGGC
		Reverse	GACAACCTTTCCCGACCCG
	Unmethylated	Forward	GTGTTGGTTGTTTGGTAGGT
		Reverse	AACAACCTTTCCCAACCCA

(B) TaqMan assays of miRNA expression assay

miRNA	TaqMan assay
miR-200a	000502
miR-200b	002251
miR-200c	002300

Supplementary Fig. S1

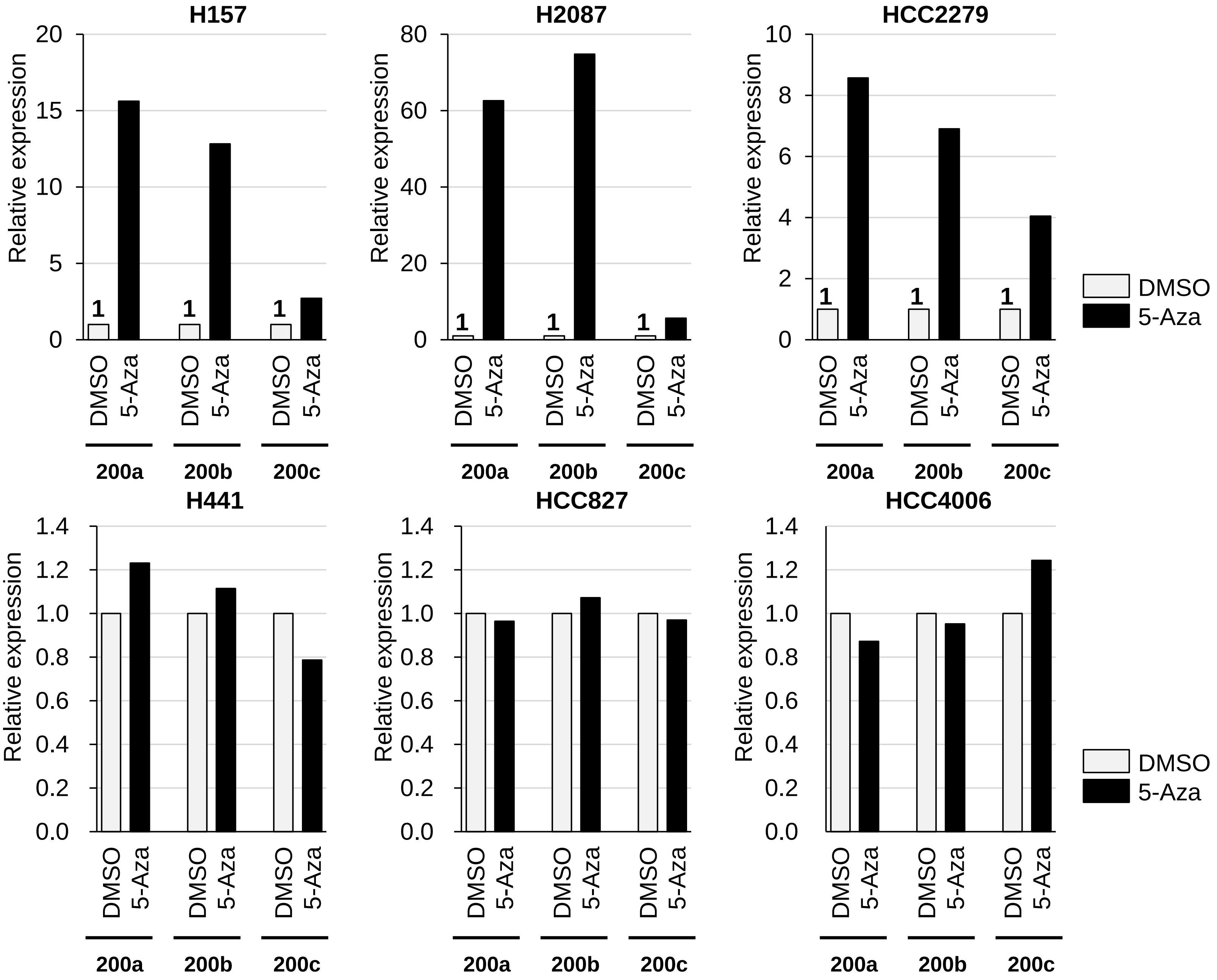

Supplementary Fig. S2

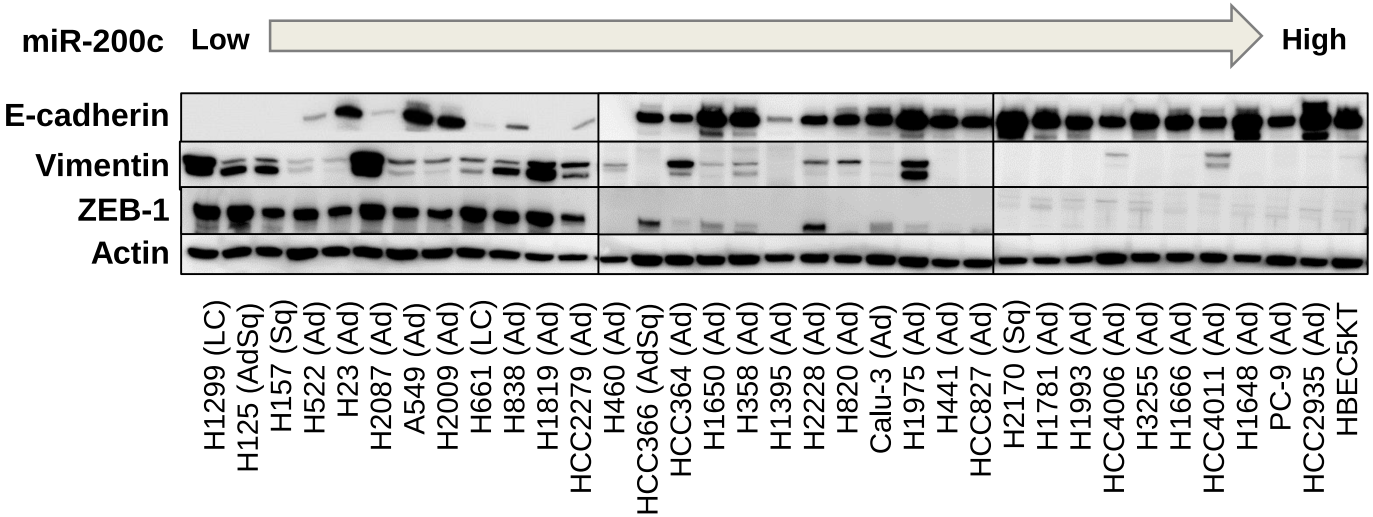

Supplementary Fig. S3

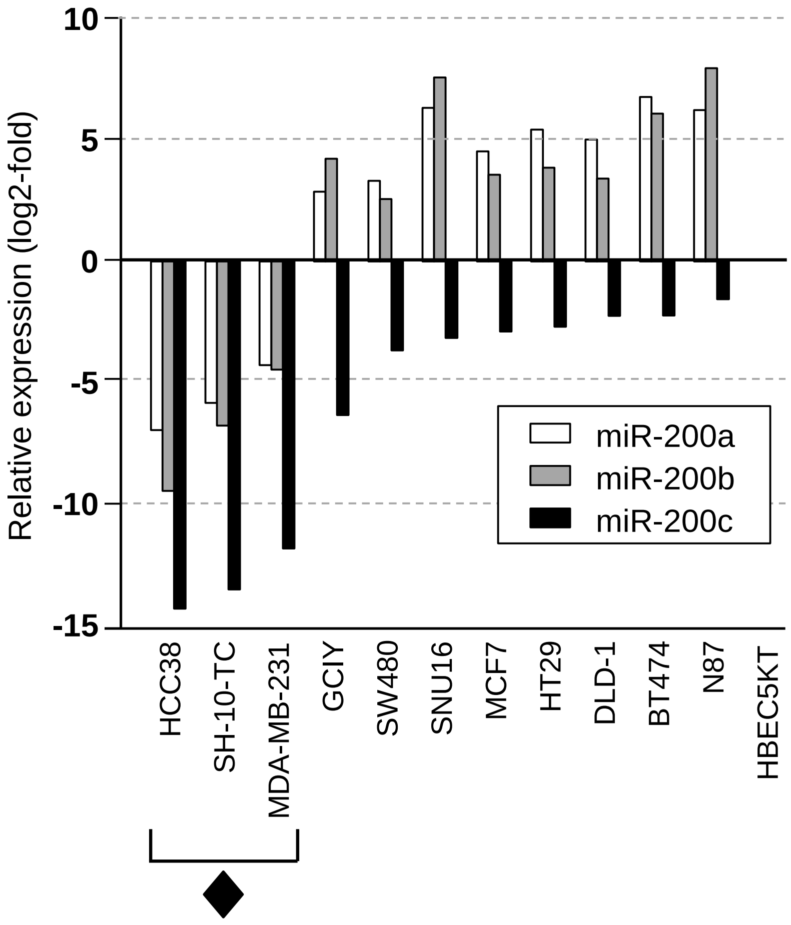

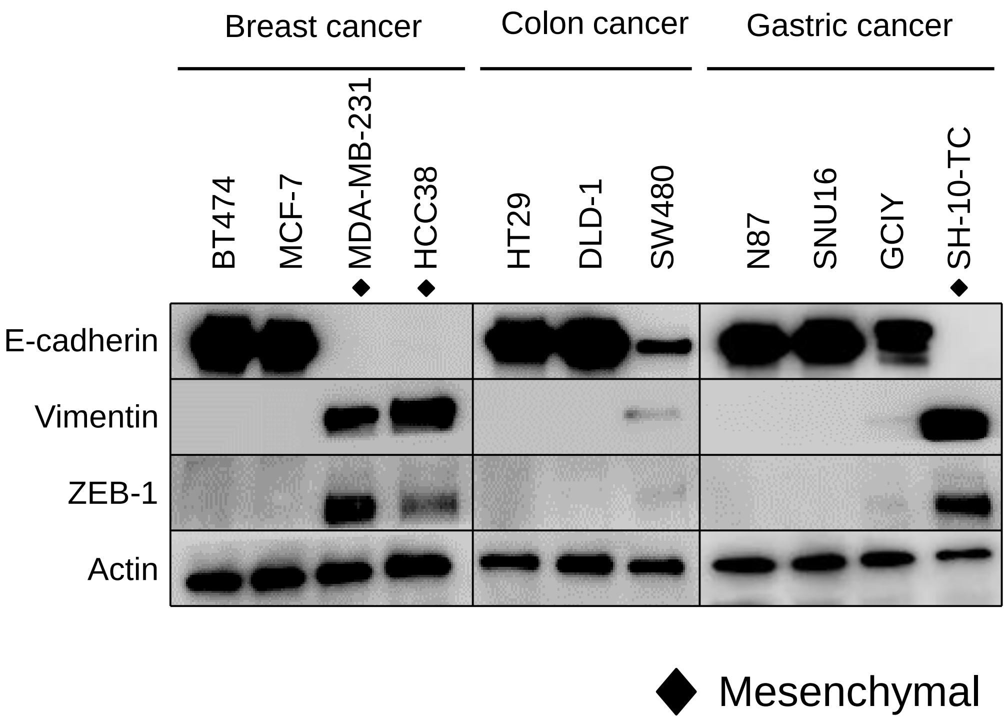

Supplementary Fig. S4

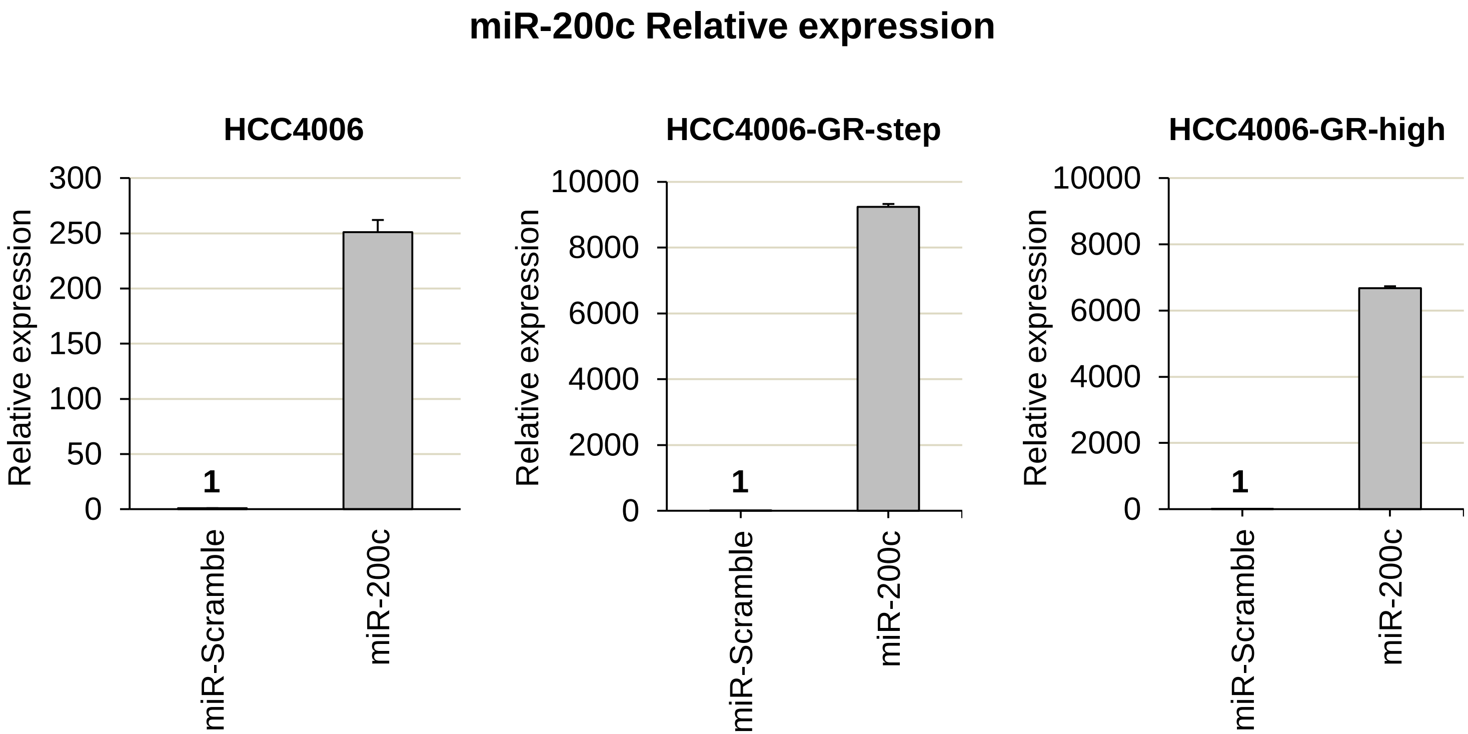

Supplementary Fig. S5

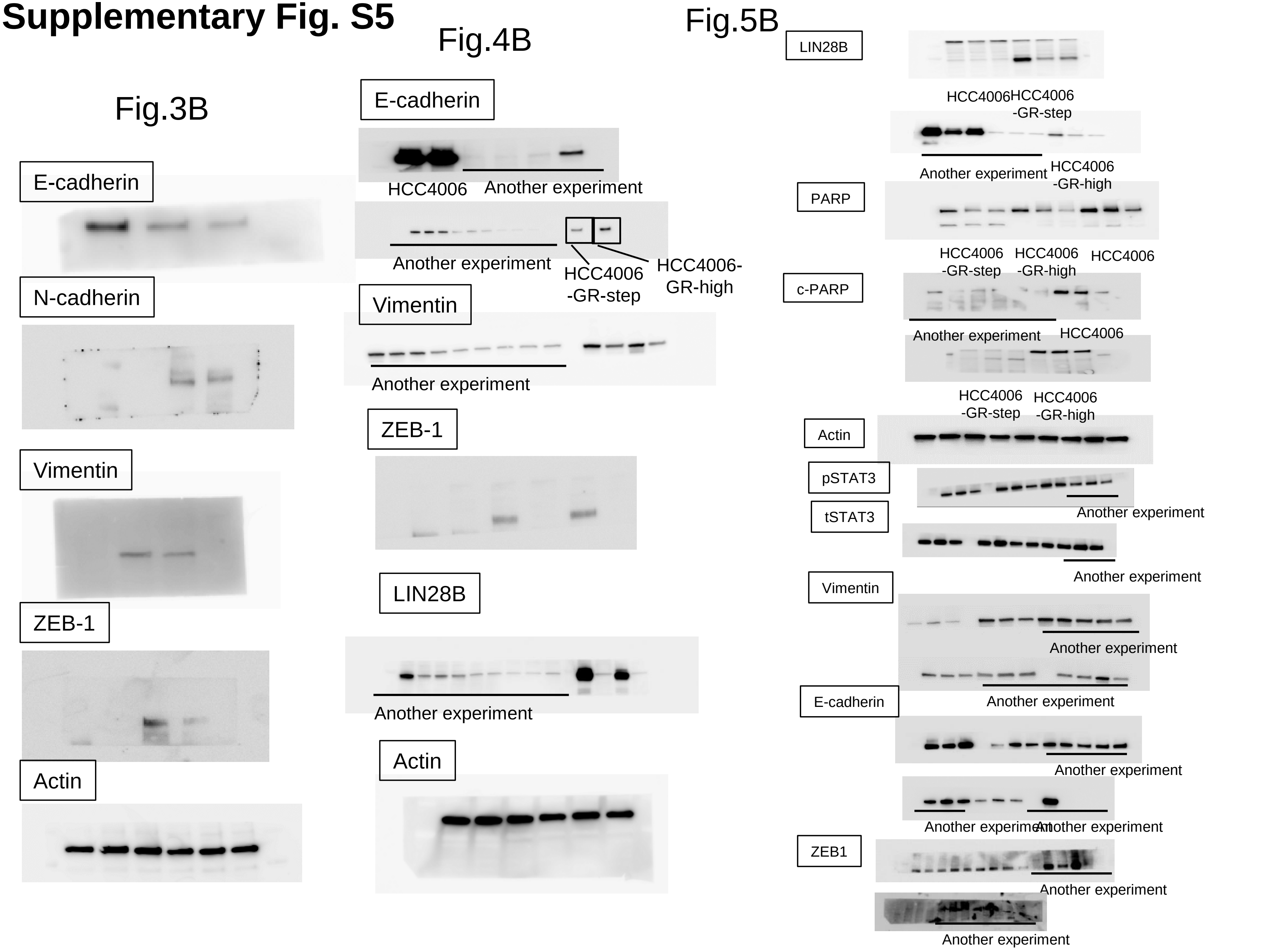

Supplement: Supplementary Information [file srep40847-s1.pdf]
